# NetSurfP-3.0: accurate and fast prediction of protein structural features by protein language models and deep learning

**DOI:** 10.1093/nar/gkac439

**Published:** 2022-06-01

**Authors:** Magnus Haraldson Høie, Erik Nicolas Kiehl, Bent Petersen, Morten Nielsen, Ole Winther, Henrik Nielsen, Jeppe Hallgren, Paolo Marcatili

**Affiliations:** Department of Health Technology, Technical University of Denmark, DK Lyngby, Denmark; Department of Health Technology, Technical University of Denmark, DK Lyngby, Denmark; Center for Evolutionary Hologenomics, GLOBE Institute, University of Copenhagen, Denmark; Centre of Excellence for Omics-Driven Computational Biodiscovery (COMBio), Faculty of Applied Sciences, AIMST University, Kedah, Malaysia; Department of Health Technology, Technical University of Denmark, DK Lyngby, Denmark; Section for Cognitive Systems, DTU Compute, Technical University of Denmark (DTU), Denmark; Center for Genomic Medicine, Rigshospitalet (Copenhagen University Hospital), Copenhagen, Denmark; Department of Biology, Bioinformatics Centre, University of Copenhagen, Copenhagen, Denmark; Department of Health Technology, Technical University of Denmark, DK Lyngby, Denmark; BioLib Technologies, Copenhagen, Denmark; Department of Health Technology, Technical University of Denmark, DK Lyngby, Denmark

## Abstract

Recent advances in machine learning and natural language processing have made it possible to profoundly advance our ability to accurately predict protein structures and their functions. While such improvements are significantly impacting the fields of biology and biotechnology at large, such methods have the downside of high demands in terms of computing power and runtime, hampering their applicability to large datasets. Here, we present NetSurfP-3.0, a tool for predicting solvent accessibility, secondary structure, structural disorder and backbone dihedral angles for each residue of an amino acid sequence. This NetSurfP update exploits recent advances in pre-trained protein language models to drastically improve the runtime of its predecessor by two orders of magnitude, while displaying similar prediction performance. We assessed the accuracy of NetSurfP-3.0 on several independent test datasets and found it to consistently produce state-of-the-art predictions for each of its output features, with a runtime that is up to to 600 times faster than the most commonly available methods performing the same tasks. The tool is freely available as a web server with a user-friendly interface to navigate the results, as well as a standalone downloadable package.

## Graphical Abstract

**Graphical Abstract:**
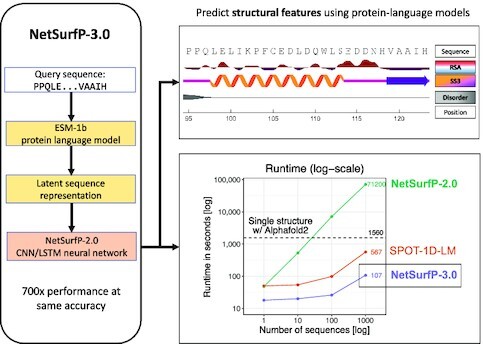
NetSurfP-3.0: accurate and fast prediction of protein structural features by protein language models and deep learning.

## INTRODUCTION

In 2020, AlphaFold demonstrated the full potential of deep-learning methods for prediction of protein structures from amino acid sequences, by achieving extraordinary results at the 14th edition of the CASP competition (1,2). As of March 2022, the pre-calculated AlphaFold database (3) contains 3D models for almost a million different proteins, substantially increasing our understanding of protein structure and function. However, while impressive, this number covers only a minute fraction of the known protein sequences, at the current time limited to <0.5% of the UniProt database (4) originating from a small group of model organisms. The bottleneck resides in the immense computational demands of running the AlphaFold model, both in terms of computing power and runtime. Therefore, there is still a need for prediction tools that can annotate protein sequences in a faster, yet accurate, manner.

One of the time-consuming steps in most of the modern tools that predict global and local structural features is the search and alignment of homologs of the target protein, usually referred to as multiple sequence alignment (MSA) (5). MSAs are used to create a compact and informative numerical representation of the residues in a collection of sequences, based on statistics derived from evolutionary-related sequences. MSAs can be generated using different tools (6–11), which vary in their precision and speed of execution. Until recently, the general paradigm has been that accurate prediction performance depends on an exhaustive search and high information content in the MSA, thus limiting the speed of such methods (12).

Recent breakthroughs in the field of natural language processing (NLP) (13) have allowed for generating information-rich representations of protein sequences without the explicit need for MSAs (14,15). This is achieved by pre-training language models (LMs) on large datasets of unannotated sequences. These LMs are trained to predict the probability of a masked residue in a protein sequence, only based on the rest of the sequence itself. In doing so, they generate complex numerical representations of protein sequences (also referred to as sequence embeddings), that can be exploited to perform downstream prediction tasks, thus bypassing the need to generate computationally expensive MSAs.

NetSurfP-2.0 is a tool that generates state-of-the-art predictions for protein secondary structure, solvent accessibility, disorder, and backbone geometry of any given protein from its primary sequence only (16). Since its launch in 2019, NetsurfP-2.0 has been used extensively by the research community to annotate proteins. The web server has received 27 456 job submissions and the stand-alone package has been downloaded 995 times. Here, we present NetSurfP-3.0, an updated version of NetSurfP, that uses the ESM-1b protein LM (14) to replace the time-consuming MSA generation used in version 2.0, thus decreasing the runtime of the tool by >2 orders of magnitude, without any substantial trade-off in terms of accuracy.

When assessed on multiple independent test sets selected to have no >25% sequence identity to any protein used in the training, its accuracy is consistently on par with or better than version 2.0 or other similar tools. At the same time, it is consistently the fastest of all the tools we tested by a large margin 1. It has a user-friendly interface allowing non-expert users to access and analyze their results via a browser thanks to its graphical output, and with its easily downloadable output in several common formats it can be used for further analysis.

**Figure 1.**
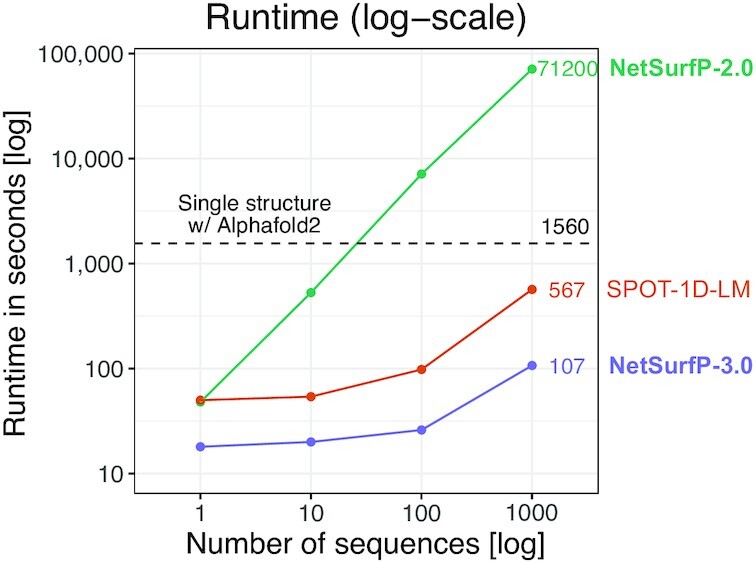
Runtime analysis in seconds for submissions of 1 to 1000 sequences for NetsurfP-3.0, SPOT-1D-LM and NetsurfP-2.0. We note a 5.3× speed-up for NetSurfP-3.0 versus its closest competitor SPOT-1D-LM, and 665× speed-up versus the older NetSurfP-2.0, when processing 1000 sequences. The runtime for a single AlphaFold2 model is shown with a dashed line. We performed all benchmarks on the same AWS EC2 G4 instance, with a 16GB NVIDIA T4 GPU (see Methods).

## MATERIALS AND METHODS

### Datasets

The NetSurfP-3.0 method has been trained and tested on the same datasets used for NetSurfP-2.0. Its training and validation set consist of 10 337 and 500 non-redundant proteins, respectively, and the testing is performed on the three test sets TS115 (115 proteins) (17), CB513 (513 protein regions from 434 proteins) (18), and CASP12 (21 proteins) (19). For an accurate description of these datasets, we refer to the original paper (16). Additionally, we tested NetSurfP-3.0 and other tools on a novel external dataset, named CASP14_FM (1). This dataset consists of all the protein domains from the CASP14 competition labelled as free modeling (FM) targets, that are targets for which no structural template, at the time of the competition, could be identified by using sequence-based homology searches. In fact, when comparing this dataset to our training and validation sets using the MMseqs2 easy-cluster tool (10), no hit with >20% sequence identity and 50% coverage was found.

### Network architecture

The NetSurfP-3.0 model and the other models used as a comparison in this paper differ in the way they encode the input sequences, while sharing the same downstream architecture of the NetSurfP-2.0 model. The previously mentioned NetSurfP-2.0 model first generates sequence profiles by utilizing either a HHBlits (9) or MMSeqs2 (10) search on the Uniref30 database (20). The probabilities extracted from these profiles are then used to encode the input sequences. The NetSurfP-2.0 sequence encoding consists of 50 variables per residue. The encoding of the baseline model, named NSP_OH, consists of the one-hot sparse encoding of each amino acid, for a total of 20 variables per residue. The NetSurfP-3.0 model exploits the embeddings generated by a pre-trained language model, ESM-1b (14). This embedding consists of 1280 variables per residue. Regardless of the embedding scheme, the embeddings are fed into two separate 1D CNN layers, using the ReLU activation function and a dropout probability of *P* = 0.5. The first 1D CNN layer has 32 output channels, kernel size of 129, and padding of size 64. The second 1D CNN layer consists of 32 output channels, a kernel size of 257, and a padding of size 128. Consequently, the output of the 1D CNNs is concatenated with the initial input, and a 1D batch normalization is applied. The output is then passed to a two-layer biLSTM with a hidden size of 1024, and a dropout probability of *P* = 0.5. The output from the last biLSTM is then fed into a fully connected layer which finally provides the outputs for the linear layers related to each of the six tasks: eight-state secondary structure (Q8), three-state secondary structure (Q3), disorder, relative solvent-accessible area (RSA), and the dihedral angles phi and psi. The Q8 output layer is configured to generate predictions for eight features, corresponding to the secondary structure classes, whereas three features are predicted in the Q3 output layer. The disorder output generated binary predictions. The RSA output generated a single feature, followed by sigmoid activation, to keep values between 0 and 1. Both phi and psi generated two features, corresponding to sine-cosine encoding of the torsion angles. The tanh activation function was applied in the prediction of phi and psi in order to maintain values between –1 and 1. Moreover, absolute solvent-accessible area (ASA) was calculated from the predicted RSA, by multiplying the RSA and ASA max. In addition to the existing NetSurfP 2.0 architecture, we investigated if other downstream architectures would improve the NetSurfP 3.0 benchmarks. The NetSurf 2.0’s CNN-LSTM downstream architecture was replaced with transformer encoder layers. The output from the ESM-1b embeddings were fed into a transformer encoder, with eight heads and two encoder layers, and used a positional encoder based on a cyclic solution. The transformer encoder output was fed into the same multitask output as in NetSurfP 2.0. Moreover, a similar approach was used by replacing the NetSurfP 2.0 LSTM only with transformer encoder layers. All the networks are implemented in PyTorch (21).

### Processing of long sequences

By default, the ESM-1b model is limited to input sequence lengths of no more than 1024 amino acids. To overcome this limitation, we use a moving window representation. For sequences exceeding the 1024 limit, we create a moving window across 1024 residues, moving with a stride of 824, corresponding to an overlap of 200 amino acids across windows. Each moving window’s sequence output representation is then trimmed by removing the first 50 and last 50 residues, and the trimmed sequence is then concatenated with the previous and next moving windows.

### Training

The validation dataset was used to perform early stopping during training. Early stopping was performed after 3 epochs in which the loss did not decrease. The training was done using mini batches of size 15. We used the default Pytorch implementation of the Adam optimizer (22), with a learning rate of 5e–4, epsilon of 1e–8, betas of 0.9 and 0.99, and zero weight decay.

Backpropagation loss was calculated using a multi-task loss function, which included a weighted loss for each task to predict the local structural features of protein structure. We used a categorical cross entropy loss function for the Q8, Q3 and disorder classification tasks, while RSA, phi and psi regression tasks used the mean squared error loss function. To keep the losses from each task even, they were weighted and summed. We normalized the weights based on the Q8 loss (which had a weight of 1 in the final total loss), with weights applied to the other losses to achieve equal influence. In order to prevent the needed padding of sequence lengths to have an impact on the loss, we masked the loss for the padded positions. RSA, phi, and psi uses additional masking for disordered regions and unknown amino acids. The weights for the different loss components were 1, 5, 5, 100, 5 and 5 for Q8, Q3, disorder, RSA, phi and psi, respectively.

### Evaluation

The models with the lowest validation loss were selected after training. When evaluating the models, the test datasets were used to predict and compare predictions, with mini batches of size 15. We used several metrics for assessing the performance of the various prediction tasks. For Q3 and Q8 secondary structure prediction, we calculated accuracy (ACC) based on the class predictions and target classes. For disorder predictions, we used the metrics false positive rate (FPR) and Matthews correlation coefficient (MCC). For relative surface accessibility (RSA), we calculated the Pearson correlation coefficient (PCC), and for phi and psi, the mean absolute error (MAE) between predictions and target values.

### Runtime analysis

Benchmarking of model runtimes was performed on the same standard Amazon web-services EC2 G4 instance with a single 16GB NVIDIA T4 GPU. Each model was given the same input FASTA file containing either 1, 10, 100 or 1000 sequences. The runtime for a single AlphaFold2 model for the single-sequence FASTA was evaluated using AlphaFold v2.0 (2) with default settings.

## RESULTS

### Model performance

We compared the performance of NetSurfP-3.0 with NetSurfP-2.0 on the original test sets used for the NetSurfP-2.0 publication. As shown in Table 2, NetSurfP-3.0 and NetSurfP-2.0 achieve similar accuracy on all datasets. As a baseline, we also include the results obtained using a simple one hot encoding strategy. The gains obtained by using either encoding derived from MSAs or LMs are evident.

**Table 1.** Performance benchmark on CASP14_fm test set proteins

Model	Q3 ↑	Q8 ↑	RSA ↑	Phi ↓	Psi ↓
	(ACC)	(ACC)	(PCC)	(MAE)	(MAE)
NetSurfP-3.0	0.601	0.607	0.599	**52.71**	42.02
NetSurfP-2.0	0.581	0.618	**0.632**	52.73	**41.37**
SPOT-1D	0.576	0.572	0.556	71.56	84.92
SPOT-1D-LM	**0.615**	**0.623**	0.601	66.64	81.72

Performance of NetSurfP-3.0, NetSurfP-2.0, SPOT-1D-Single (SPOT-1D) and SPOT-1D-LM (SPOT-1D-LM) on the CASP14_FM dataset. Each column represents a different output variable, with the associated metric. Up- and down-facing arrows indicate metrics for which an improvement is represented by larger or lower values, respectively. The values corresponding to the best performances are in bold.

**Table 2.** Model performance when applying one-hot encodings or ESM1b embeddings to predict protein local structure

Test dataset	Model	RSA ↑	ASA ↑	Q8 ↑	Q3 ↑	Disorder ↑	Disorder ↓	Phi ↓	Psi ↓
		(PCC)	(PCC)	(ACC)	(ACC)	(MCC)	(FNR)	(MAE)	(MAE)
CB513	NSP One-Hot	0.628	0.669	0.573	0.719	-	-	25.80	46.16
	NetSurfP-2.0	0.791	0.804	**0.713**	0.845	-	-	20.35	**29.04**
	NetSurfP-3.0	**0.793**	**0.810**	0.711	**0.846**	-	-	**20.22**	29.25
TS115	NSP One-Hot	0.633	0.679	0.628	0.746	0.561	**0.006**	22.60	41.40
	NetSurfP-2.0	0.771	0.793	0.740	0.849	0.624	0.013	17.40	26.80
	NetSurfP-3.0	**0.776**	**0.799**	**0.749**	**0.856**	**0.662**	0.015	**17.16**	**25.80**
CASP12	NSP One-Hot	0.570	0.608	0.576	0.704	0.573	**0.007**	26.30	46.70
	NetSurfP-2.0	**0.728**	**0.739**	**0.699**	**0.810**	**0.653**	0.015	**20.90**	**32.80**
	NetSurfP-3.0	0.707	0.722	0.669	0.791	0.621	0.024	21.25	33.92

Assessment of NetSurfP-3.0, NetSurfP-2.0, and NetSurfP with one-hot encoding (NSP One-Hot) on the CB513, TS115 and CASP12 datasets. Each column reports an output variable with the corresponding metrics: Pearson correlation coefficient (PCC), accuracy (ACC), Matthews correlation coefficient (MCC), false positive rate (FPR) and mean absolute error (MAE). Up- and down-facing arrows indicate metrics for which an improvement is reprepresented by larger or lower values, respectively. For each dataset, the values corresponding to the best performances are in bold.

A marginal decrease in performance is observed on the CASP12 dataset; it should be noted that, because of the limited size of this dataset, such an effect might be due to statistical fluctuations in training and assessment. From these results, it appears that the ESM-1b encoding can successfully replace the MSA giving a similar accuracy in performance, while at the same time giving a dramatic increase in speed, hence allowing for large-scale analyses on the proteome level. Interestingly, we tried to replace the NetSurfP-2.0 downstream architecture by the transformer-based architectures described in the methods, and in all cases, when using ESM-1b encodings, we obtained sub-optimal performances. We also tested these models, together with other available similar models, on the new dataset CASP14_FM. The results are shown in Table 1.

The models compared are NetSurfP version 2.0 and 3.0, Spot-1D-Single (23) and SPOT-1D-LM (24). Both SPOT-1D-Single and SPOT-1D-LM do not make use of MSAs in their prediction. Moreover, SPOT-1D-LM uses a combination of different protein LMs to encode the target sequences, thus making it the closest comparison to NetSurfP-3.0. From these results, it appears that NetSurfP-3.0 generates results that are on par with the other tools for secondary structure and solvent accessibility. In particular, when compared with SPOT-1D-LM, it shows better results for phi and psi prediction. Given the similar accuracy displayed by the best performing tools, i.e NetSurfP-2.0, NetSurfP-3.0 and Spot1d-LM, we have also compared such tools in terms of their runtime. The results are shown in Figure 1.

NetSurfP-3.0 is consistently the fastest tool, being able to generate predictions for a thousand proteins in ∼100 s. From the runtime analysis, we see that the two fastest models are NetSurfP-3.0 and SPOT-1D-LM, with the former being 3.8–5.3 times faster than the latter. Both of these tools, after the initial overhead due to the language models being loaded into memory, scale very gradually on larger datasets, and their performance is mainly limited by the amount of memory on the device used for predictions.

### Interface

**Figure 2.**
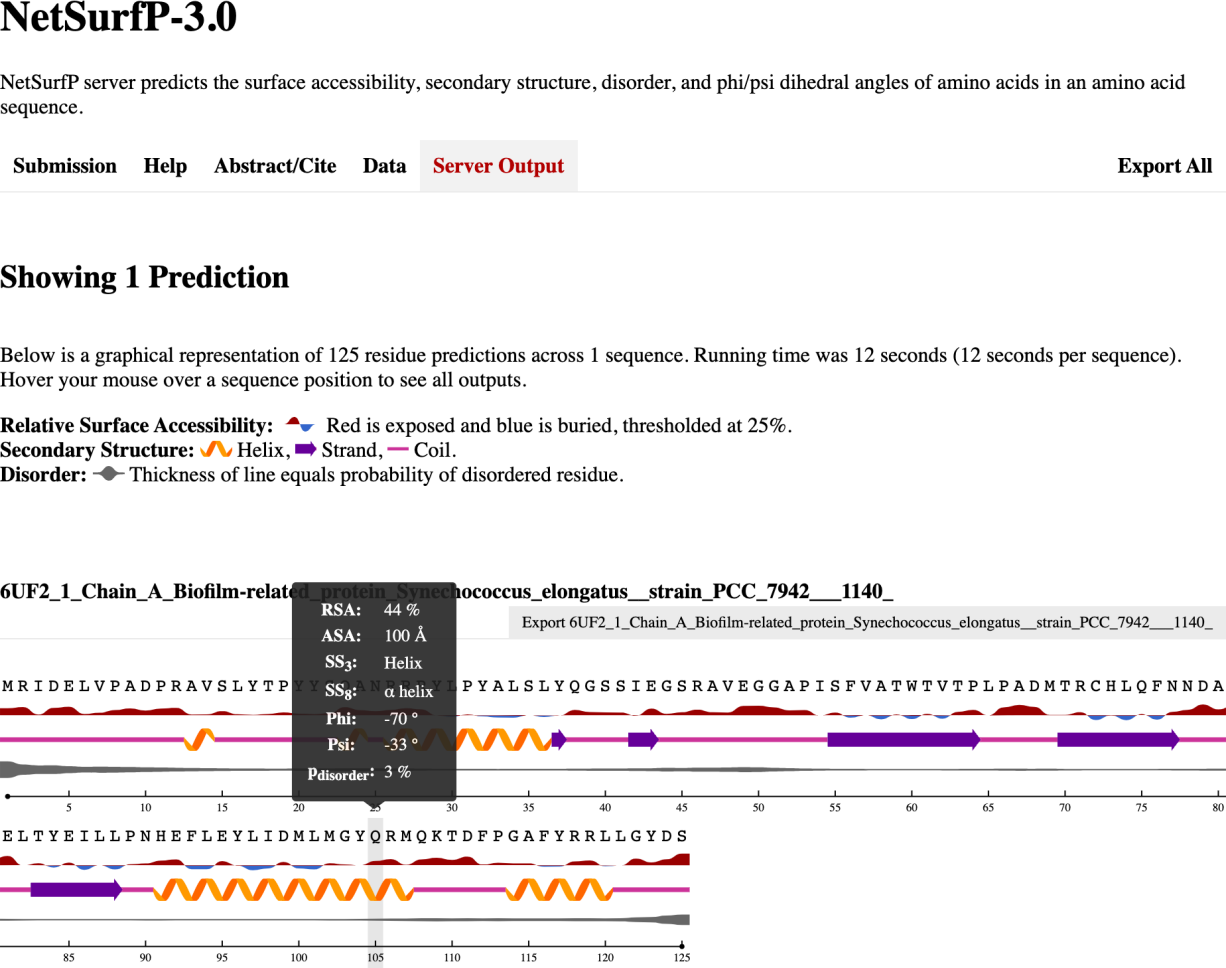
The web interface of NetSurfP-3.0. A graphical representation displays Q3 secondary structure, RSA, and disorder predictions together with the sequence. By hovering on a residue, the user can visualize all predictions. Through the Export menus it is possible to download individual predictions in different file formats, as well as the complete results as a compressed archive.

NetSurfP-3.0 can be accessed via a web server. It is sufficient to input the target sequences in FASTA format, either in the text box or upload as a file. Once the job is submitted, it enters a queue. Once the results are ready, they are displayed in the browser. An example of the output is reported in Figure 2. For each protein, the predictions are displayed in an easily understandable format, and the individual numeric predicted values can be observed by hovering the cursor over individual residues in the plot. Finally, the results can also be downloaded in different formats, either individually or as a compressed archive for all the predictions.

## DISCUSSION

The NetSurfP-3.0 web server provides state-of-the-art sequence-based predictions for solvent accessibility, secondary structure, disorder, and backbone geometry at an unprecedented speed. This is made possible by the pre-trained ESM-1b language model used to encode the proteins and by replacing the MSAs used in the previous version while retaining similar accuracy. This allows for predictions on complete proteomes in less than an hour. Other works (24) combine the embeddings from different LMs (14)(15), thus obtaining a small increment in accuracy, at the price of a slower run time. While we have focused primarily on the time efficiency of our tool, we believe that, as new and more elaborate LMs are released, it will be of utter importance to test and compare their ability to provide powerful representations of protein sequences for different downstream tasks.

Our tool is available both as a stand-alone package and as a web server. The web server, thanks to its informative and easy interface, allows the users to access and analyse their results in an intuitive and thorough way.

## DATA AVAILABILITY

NetSurfP-3.0 is available both as a web server, and as stand-alone software including datasets at the below URLs. The web server version accepts up to 10 000 sequences with lengths between 10 and 5000 residues each. The maximum number of residues in a job can not exceed 10 000 000.

- Webserver, DTU: *https://services.healthtech.dtu.dk/service.php?NetSurfP-3.0*
- Webserver, BioLib: *https://dtu.biolib.com/nsp3*
